# Grey matter networks in women and men with dementia with Lewy bodies

**DOI:** 10.1038/s41531-024-00702-5

**Published:** 2024-04-13

**Authors:** Annegret Habich, Javier Oltra, Christopher G. Schwarz, Scott A. Przybelski, Ketil Oppedal, Anna Inguanzo, Frédéric Blanc, Afina W. Lemstra, Jakub Hort, Eric Westman, Barbara Segura, Carme Junque, Val J. Lowe, Bradley F. Boeve, Dag Aarsland, Thomas Dierks, Kejal Kantarci, Daniel Ferreira

**Affiliations:** 1 Division of Clinical Geriatrics, Center for Alzheimer Research, Department of Neurobiology, Care Sciences and Society, Karolinska Institute, Stockholm, Sweden; 2 University Hospital of Psychiatry and Psychotherapy Bern, University of Bern, Bern, Switzerland; 3 Medical Psychology Unit, Department of Medicine, Institute of Neurosciences, University of Barcelona, Barcelona, Spain; 4 Institute of Biomedical Research August Pi i Sunyer (IDIBAPS), Barcelona, Spain; 5 Department of Radiology, Mayo Clinic, Rochester, MN USA; 6 Quantitative Health Sciences, Mayo Clinic, Rochester, MN USA; 7 Department of Electrical Engineering and Computer Science, University of Stavanger, Stavanger, Norway; 8 Day Hospital of Geriatrics, Memory Resource and Research Centre (CM2R) of Strasbourg, Department of Geriatrics, Hopitaux Universitaires de Strasbourg, Strasbourg, France; 9 ICube Laboratory and Federation de Medecine Translationnelle de Strasbourg (FMTS), University of Strasbourg and French National Centre for Scientific Research (CNRS), Team Imagerie Multimodale Integrative en Sante (IMIS)/ICONE, Strasbourg, France; 10 Department of Neurology and Alzheimer Center, VU University Medical Center, Amsterdam, Netherlands; 11 Memory Clinic, Department of Neurology, Second Faculty of Medicine, Charles University, Prague, Czech Republic; 12 Motol University Hospital, Prague, Czech Republic; 13 Department of Neuroimaging, Centre for Neuroimaging Sciences, Institute of Psychiatry, Psychology and Neuroscience, King’s College London, London, UK; 14 Centro de Investigación Biomédica en Red Enfermedades Neurodegenerativas (CIBERNED: CB06/05/0018-ISCIII), Barcelona, Catalonia Spain; 15 Department of Neurology, Mayo Clinic, Rochester, MN USA; 16 Department of Old Age Psychiatry, Institute of Psychiatry, Psychology & Neuroscience, King’s College London, London, UK; 17 Center for Age-Related Medicine, Stavanger University Hospital, Stavanger, Norway; 18 Facultad de Ciencias de la Salud, Universidad Fernando Pessoa Canarias, Las Palmas, Spain

**Keywords:** Dementia, Neurodegeneration, Movement disorders, Neurodegenerative diseases

## Abstract

Sex differences permeate many aspects of dementia with Lewy bodies (DLB), yet sex differences in patterns of neurodegeneration in DLB remain largely unexplored. Here, we test whether grey matter networks differ between sexes in DLB and compare these findings to sex differences in healthy controls. In this cross-sectional study, we analysed clinical and neuroimaging data of patients with DLB and cognitively healthy controls matched for age and sex. Grey matter networks were constructed by pairwise correlations between 58 regional volumes after correction for age, intracranial volume, and centre. Network properties were compared between sexes and diagnostic groups. Additional analyses were conducted on *w*-scored data to identify DLB-specific sex differences. Data from 119 (68.7 ± 8.4 years) men and 45 women (69.9 ± 9.1 years) with DLB, and 164 healthy controls were included in this study. Networks of men had a lower nodal strength compared to women. In comparison to healthy women, the grey matter networks of healthy men showed a higher global efficiency, modularity, and fewer modules. None of the network measures showed significant sex differences in DLB. Comparing DLB patients with healthy controls revealed global differences in women and more local differences in men. Modular analyses showed a more distinct demarcation between cortical and subcortical regions in men compared with women. While topologies of grey matter networks differed between sexes in healthy controls, those sex differences were diluted in DLB patients. These findings suggest a disease-driven convergence of neurodegenerative patterns in women and men with DLB, which may inform precision medicine in DLB.

## Introduction

Traditionally, dementia with Lewy bodies (DLB) has been considered a male-predominant disease. Supporting this notion, autopsy results showed a predominance of men dying with α-synuclein related pathology^1^. However, the sex distribution of DLB patients diagnosed clinically is more ambiguous. While most European and North American cohorts are dominated by men with DLB^2–4^, a systematic review on clinical studies as well as French and Chinese cross-sectional studies questioned the male predominance in DLB^5–7^.

Several studies demonstrated that differences in women and men with DLB permeate numerous aspects of the disease presentation. In that regard, women showed more widespread reductions in dopaminergic activity compared to men with DLB^8^, indicating sex differences in the spread of the disease through the brain. The hypothesis of a more aggressive disease course in women with DLB was supported by a recent in-vivo study^9^. Therein, women with DLB exhibited more abnormal concentrations of α-synuclein in cerebrospinal fluid, were older, had a shorter duration of cognitive complaints, and displayed more psychiatric and cognitive symptoms than men with DLB. Additionally, sex differences have been found regarding core clinical features of DLB and their order of appearance^10^. Whereas men with DLB more often present with REM-sleep behaviour disorder (RBD) and parkinsonism^11,12^, visual hallucinations are typically more frequent in women with DLB^12,13^. The sex differences in clinical features and their time course, together with a higher likelihood of Alzheimer’s disease co-pathology in women, were previously suggested to cause a delay in women meeting clinical diagnostic criteria for DLB^9,10,14,15^.

Currently, there is no available topographical biomarker for the spread of α-synuclein pathology in the brain in-vivo. Instead, potential sex differences in α-synuclein pathology spread could be indirectly assessed through the brain network by the impact of pathology on distinct brain regions using MRI. Specifically, higher levels of atrophy have been observed in regions surrounding the substantia nigra^16^, which is hypothesized to be affected early by the α-synuclein pathology. However, regional analyses do not appreciate the hypothesized transneuronal spread of the α-synuclein pathology through the brain network and its accumulation in an increasing number of brain regions^17^. Instead, associations between affected brain regions can be revealed via the assessment of the brain network with graph-theoretical approaches. Using such a network approach, a previous study found differences in large-scale structural grey matter networks between DLB patients and healthy controls, yet that study did not investigate sex differences^18^. In contrast, three MRI studies, one of them using the same cohort as the current study, investigated sex differences in regional and lobar atrophy but they did not inspect grey matter networks^19–21^. In these studies, men with DLB had a greater grey matter loss compared to women with DLB, especially in frontal regions and at younger ages^21^. However, sex differences in grey matter networks of DLB patients remain to be investigated.

The current study had three aims. First, we assessed sex differences in grey matter network topologies in DLB patients using graph theoretical analyses on regional volumetric measures from structural MRI. The findings in DLB patients were assessed in relation to sex differences in grey matter network topologies in healthy elderly controls. Second, we aimed to extract DLB-specific sex differences in grey matter networks. To do that, we constructed grey matter networks on *w*-scored DLB data, thus removing sex differences found in healthy elderly controls and disentangle them from disease-related sex differences. Third, to resolve whether disease-related changes in women or men drive DLB-specific sex differences in grey matter networks, we compared DLB patients and healthy controls within both sexes. We hypothesised that sex differences in network topologies differ between DLB patients and healthy controls, as structural brain deterioration in DLB may redirect sex-specific volume reductions experienced during normal ageing.

## Results

### Cohort characteristics

As expected, DLB patients (*n* = 164, 45 women) and healthy controls (*n* = 164, 45 women) differed in their MMSE scores, with DLB patients showing lower cognitive performance (Table 1). DLB patients and healthy controls also differed in years of education, with healthy men having received a longer education. In the DLB patient group, men were more likely to exhibit parkinsonism than women, with none of the other core features showing sex differences. There were no significant sex differences in disease duration, MMSE, or AD co-pathology between women and men with DLB.

**Table 1:** Demographic and clinical characteristics of DLB patients and healthy controls

Table	DLB Patients (*n* = 164)		Healthy Controls (*n* = 164)		Statistics
Variables	Women (*n* = 45)	Men (*n* = 119)	Women (*n* = 45)	Men (*n* = 119)	
Age (years)	69.9 ± 9.1	68.7 ± 8.4	69.9 ± 9.1	68.7 ± 8.4	*F*_(327,3)_ = 0.4*, p* = 0.757 (ANOVA)
Education (years)	13.4 ± 3.5	13.7 ± 4.0	14.4 ± 2.3	15.3 ± 2.7	*F*_(327,3)_ = 6.13, *p* < 0.001 (ANOVA) women_DLB_, men_DLB _< men_HC_
Disease duration (years)	4.8 ± 3.1	5.8 ± 4.6	n.a.	n.a.	*t*_(120)_ = −1.03, *p* = 0.305 (*t*-test)
MMSE	24.4 ± 4.2	22.3 ± 5.5	28.6 ± 1.1	28.4 ± 1.3	*F*_(327,3)_ = 63.42, *p* < 0.001 (ANOVA) women_DLB_, men_DLB_ < women_HC_, men_HC_
TIV	1439.8 ± 117.7	1632.0 ± 135.0	1391.0 ± 107.3	1611.8 ± 124.4	*F*_(327,3)_ = 60.69, *p* < 0.001 (ANOVA) women_DLB_ women_HC_ < men_DLB_, men_HC_
Parkinsonism	35 (77.8%)	106 (90.6%)^a^	n.a.	n.a.	*p* = 0.038 (Fisher’s test)
Visual hallucinations	27 (60.0%)	62 (53.4%)^b^	n.a.	n.a.	*p* = 0.484 (Fisher’s test)
Cognitive fluctuations	36 (81.8%)^c^	94 (83.9%)^d^	n.a.	n.a.	*p* = 0.812 (Fisher’s test)
Probable RBD	27 (71.8%)^d^	89 (80.2%)^e^	n.a.	n.a.	*p* = 0.262 (Fisher’s test)
AD co-pathology	4 (10.8%)^e^	9 (10.6%)^f^	n.a.	n.a.	*p* = 0.753 (Fisher’s test)

For continuous variables, data is provided as mean ± standard deviation. For categorical variables, count and percentage are provided. Missing data for ^a^*n* = 2, ^b^*n* = 3, ^c^*n* = 1, ^d^*n* = 7, ^e^*n* = 8, ^f^*n* = 34 DLB patients. ANOVA for all four groups, *t*-test or Fisher’s exact test for comparisons between women and men with DLB. ANOVAs showing a significant group effect were followed up with post-hoc *t*-tests. Results that survived the Bonferroni correction are presented in the table.

*AD* Alzheimer’s disease, *MMSE* Mini Mental State Examination, *TIV* total intercranial volume, *RBD* REM sleep behaviour disorder.

### Sex differences in grey matter network measures of healthy controls and DLB patients

**Fig. 1:**
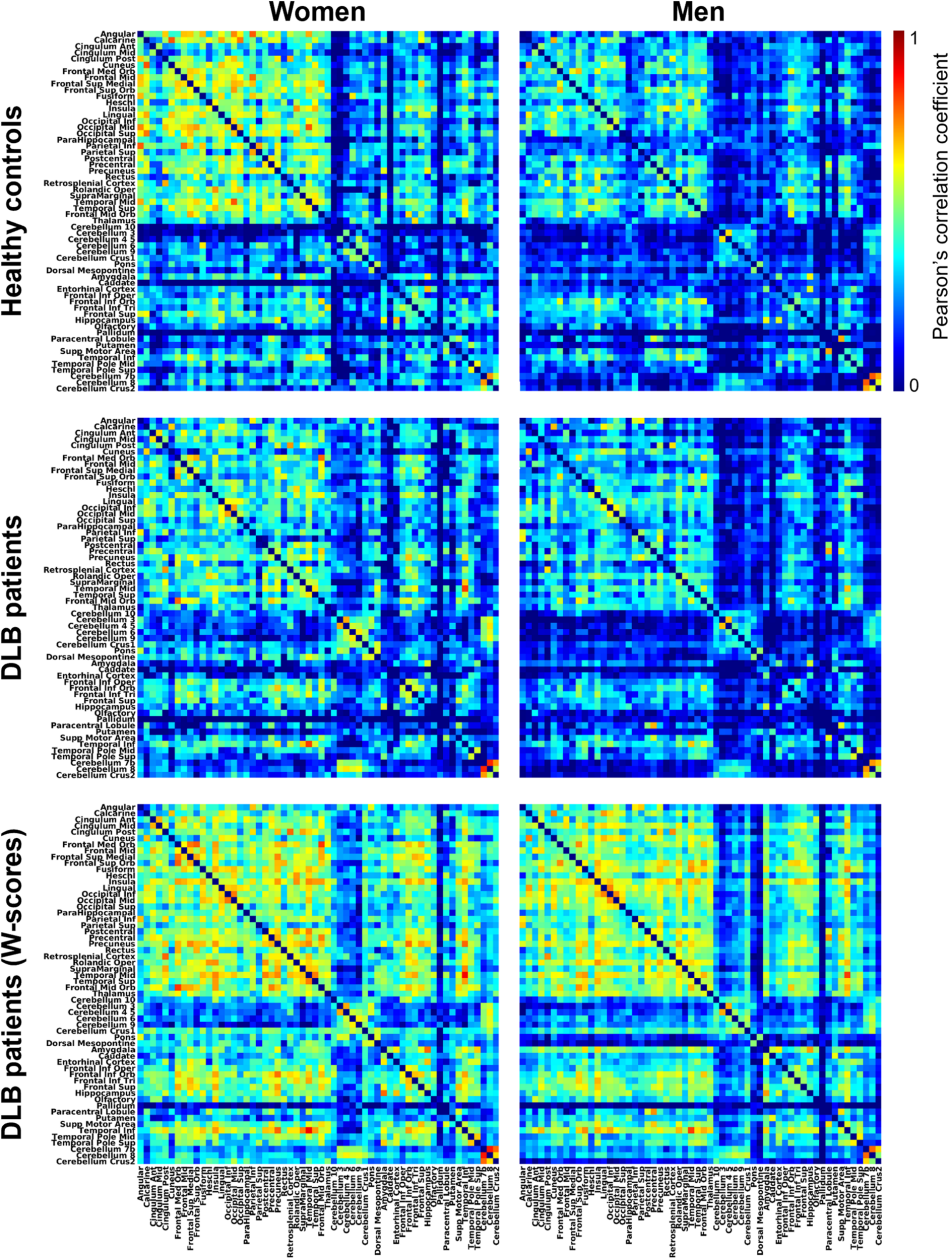
Grey matter networks. Weighted correlation matrices for women and men healthy controls, DLB patients and *w*-scored data of DLB patients. DLB dementia with Lewy bodies.

**Fig. 2:**
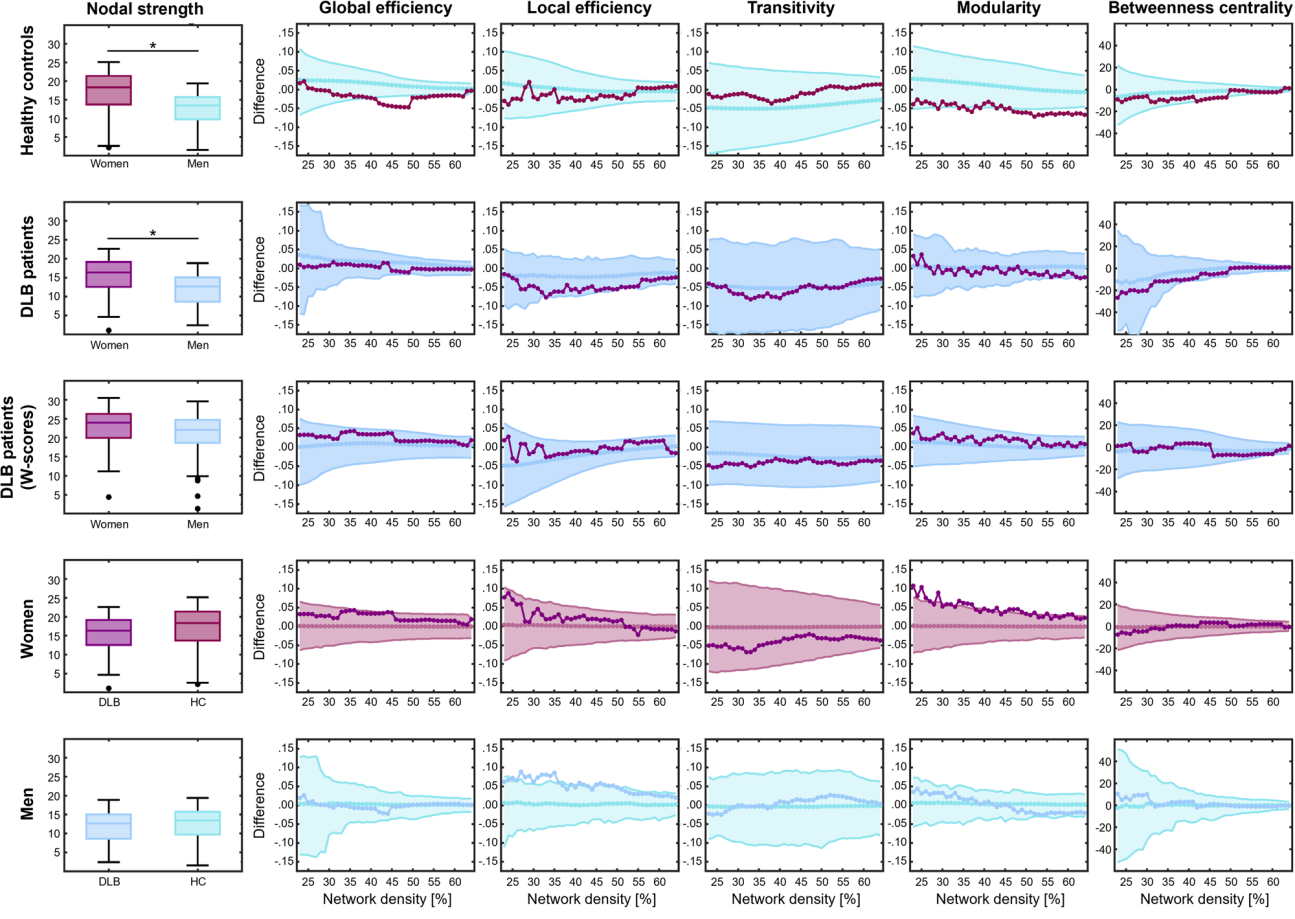
Comparison of global network measures. For nodal strength, box limits denote 25th and 75th percentiles, while whiskers indicate extreme data points without outliers. The central line denotes the median. For the remaining network measures, grey matter network densities are displayed on the *x*-axis from min = 23% to max = 64%, in steps of 1%. Group differences are displayed on the *y*-axis with 95% confidence intervals of 10’000 permutations. Negative differences indicate lower value in women or DLB patients compared to men or healthy controls, respectively. Positive differences indicate higher values in women or DLB patients compared to men or healthy controls, respectively. DLB dementia with Lewy bodies, HC healthy controls.

First, we addressed aim 1 of our study, investigating sex differences in grey matter network topologies of DLB patients and healthy controls. Visual inspection of the weighted correlation matrices (Fig. 1) showed a more sparsely connected structural network in both men with DLB and healthy men compared to their respective female counterparts. This is reflected in the lower nodal strength in men compared to women with DLB (*t*_(114)_ = 4.28, *p* < 0.001) as well as in healthy men compared to healthy women (*t*_(114)_ = 4.52, *p* < 0.001). In healthy controls, sex differences emerged in global network measures. Men showed a higher global efficiency and higher modularity (Fig. 2). No sex differences emerged in local efficiency, transitivity, and betweenness centrality in healthy controls. In contrast, none of the global measures differed significantly between sexes in DLB patients. None of the sex differences in nodal network measures survived FDR-adjustment for any node in both healthy controls and DLB patients.

Second, we aimed to pinpoint DLB-specific sex differences in grey matter networks based on *w*-scores. DLB-specific grey matter networks did not significantly differ between sexes in any of the global or nodal network measures (Fig. 2).

Third, we aimed to resolve whether men or women drive the DLB-specific sex differences in grey matter networks by comparing DLB patients to healthy controls. Our analyses revealed that women with DLB showed a higher modularity compared to healthy women, whereas men with DLB showed a higher local efficiency than healthy men. None of the remaining comparisons of global measures reached statistical significance.

Sensitivity analyses after removing the four least connected nodes (caudate, pallidum, putamen, and MCALT atlas region 10 of cerebellum) showed comparable results to main analyses (Supplementary material, Fig. 1).

### Grey matter modules of healthy controls and DLB patients

**Fig. 3:**
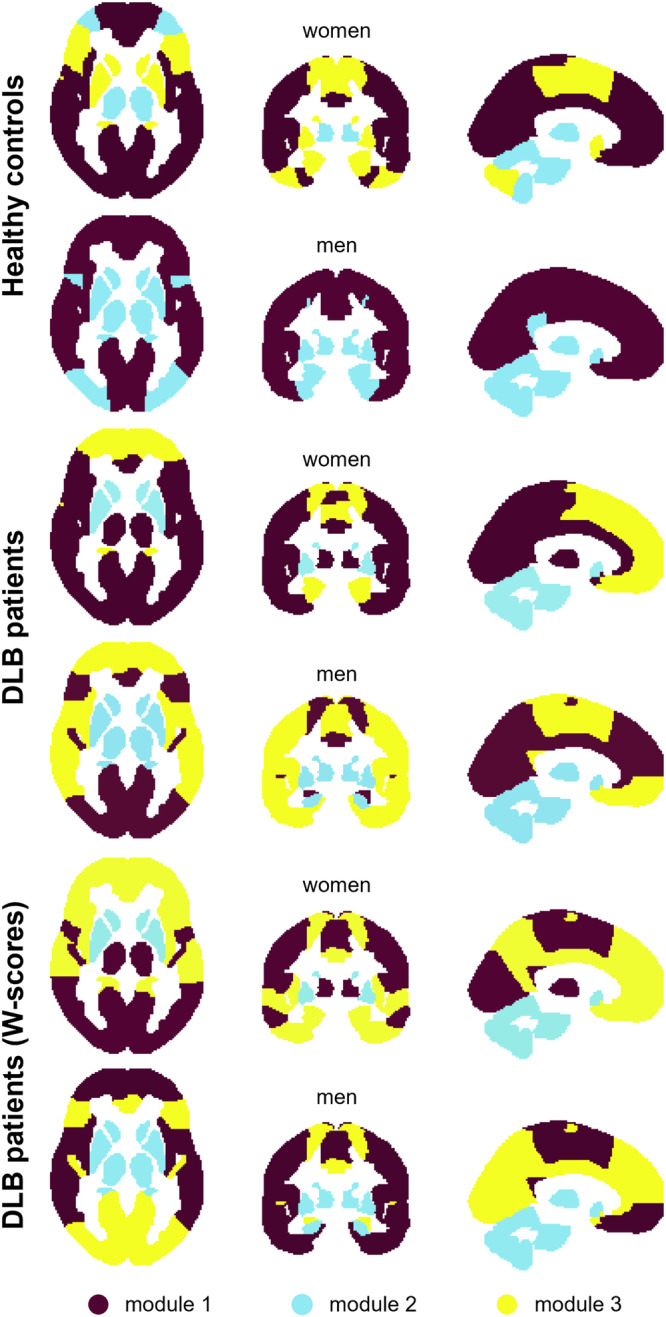
Modular organisation of grey matter networks. Modules of women and men healthy controls, DLB patients, and *w*-scored data of DLB patients. DLB dementia with Lewy bodies.

The sex-specific modules of healthy controls and DLB patients are illustrated in Fig. 3. A list of brain regions in each module is provided in Supplementary Table 1.

The grey matter network of healthy women split into 3 modules. Module I covered large parts of the cortex. In contrast, module II included only one cortical region, the middle orbital frontal gyrus, in combination with thalamus, the two pontine regions, and many cerebellar regions. Module III consisted of the remaining cortical regions, almost all subcortical regions (except thalamus), as well as the remaining three cerebellar regions. Of note, healthy women were the only group in which cerebellar regions were assigned to more than one module. Healthy men were the only group with only two modules. Module I included exclusively cortical regions. Module II combined the remaining cortical regions with all subcortical, cerebellar, and pontine regions. In sum, modules in healthy women were more fragmented whereas modules in healthy men replicated the distinction between cortical and non-cortical brain regions.

In women with DLB, module I encompassed most cortical regions, while also including thalamus, and dorsal mesopontine. Module II spanned subcortical regions (caudate, pallidum, putamen), all cerebellar regions, pons, and one cortical region. Module III encompassed the hippocampus and remaining cortical regions. In men with DLB, the grey matter network was also split into three modules. Module I covered many cortical regions and the amygdala. Module II was an almost exclusively non-cortical module, including most subcortical, all cerebellar, and pontine regions. Lastly, module III included the remaining cortical regions and insula. Taken together, visual inspection of modules in DLB patients showed that cortical regions largely clustered together in both women and men. Likewise, non-cortical regions mostly clustered together while being separate from cortical regions in both sexes.

Grey matter networks calculated on *w*-scores of both women and men with DLB were divided into 3 modules (Fig. 3), with module affiliation being similar between the sexes. In women with DLB, module I combined cortical regions and thalamus. Module II included several subcortical, all cerebellar and pontine regions as well as the angular gyrus. The largest module III spanned all the remaining cortical regions, amygdala, and hippocampus. In men with DLB, module I incorporated exclusively cortical regions. Module II comprised most subcortical regions, in combination with all cerebellar, and pontine regions. In Module III, amygdala and cingulum clustered together with the remaining cortical regions. In summary, module assignments in w-scores showed a greater match between women and men with DLB compared to healthy women and men.

## Discussion

In this study, we showed that sex differences in grey matter network topologies materializing in healthy elderly individuals were diluted in DLB patients.

In the first step, we evaluated sex differences in grey matter networks in healthy elderly adults as a basis of comparison for sex differences in DLB patients. Our analyses showed a lower network strength in healthy men, in combination with a higher global efficiency, higher modularity, and lower number of modules in grey matter networks in healthy men compared to healthy women. Taken together, these network differences point towards a weaker but more integrated and less segregated grey matter network in healthy men, which indicates a more widespread pattern of neurodegeneration in healthy men compared to healthy women. This is in line with previous reports of age-related grey matter atrophy starting earlier and proceeding at a faster rate in healthy men compared to healthy women^22,23^. Sex differences in age-related neurodegeneration have been shown to occur in various cortical and subcortical brain regions, with atrophy starting earlier or proceeding at a faster rate in men^24,25^. Given the early involvement of subcortical structures in the spread of the α-synuclein pathology^26^, the age-related neurodegeneration in those regions may ultimately drive the higher vulnerability of men to DLB. In turn, this may drive the higher prevalence of DLB in men in most European and North American cohorts and the earlier diagnosis of DLB in men^2–4,10,14^.

In contrast, our analyses on *w*-scores showed that none of the observed sex differences in network measures in healthy controls were statistically significant in DLB patients. At a first glance, this might seem to be at odds with the previously reported sex differences in many aspects of DLB, ranging from epidemiology to pathogenesis to progression to symptom manifestation^2–4,9,12,27^. However, our findings suggest a potentially disease-driven convergence of grey matter networks in women and men with DLB. This may be driven by a late and more disruptive effect of the disease on the grey matter networks in women with DLB whereas grey matter networks in men with DLB reach the extreme degree of network disorganization earlier in the disease. This aligns with the convergence of regional atrophy measures in women and men with DLB with increasing age as revealed by our previous study in the same cohort^21^.

It also needs to be considered that the hypothesized transneuronal spread of the α-synuclein pathology^17^ requires connections between brain regions for the progression of the α-synuclein pathology through the brain network as previously shown in Parkinson’s disease and idiopathic RBD^27,28^. Our recent systematic review revealed widespread network disruptions across a variety of neuroimaging methods in DLB that potentially precede regional atrophy^29^. To the best of our knowledge, there is only a single study investigated sex differences in metabolic networks, which showed more widespread connectivity disruptions in men with DLB^30^. The observed disruptions might stall the spread of the α-synuclein pathology in men with DLB, allowing the spread to catch up in women with DLB, eventually leading to a convergence of the associated neurodegeneration. Similarly, our recent study suggested that while women with DLB show less atrophy at younger ages compared with men with DLB, these differences disappear at older ages due to faster atrophy rates in women with DLB^21^. The diminished female advantage at older age has been related to the loss of the neuroprotective effect of female sex hormones during menopause^31^. Previous studies suggested that the accelerated deterioration of brain structure and function in women as compared to men was particularly pronounced in the advent of prodromal and clinical Alzheimer’s disease^32,33^. Similarly, an increased vulnerability of post-menopausal women to pathologies may apply to DLB and Parkinson’s disease for which previous studies reported a dilution of sex differences in functional connectivity measures and global topologies of resting-state networks^34,35^.

In our study we made a point of including non-cortical ROIs in addition to cortical ROIs. According to currently applied staging systems, the α-synuclein pathology originates in the brain stem, olfactory bulb, or amygdala from where it spreads to limbic structures before reaching neocortical regions at Braak stage IV^26,36^. In turn, the regional accumulation of α-synuclein pathology has been connected to neurodegeneration in the affected brain region^37^, emphasizing the importance of including regions with early accumulation of α-synuclein in our grey matter networks, such as brain stem and subcortical regions. Since previous studies pointed to an accumulation of α-synuclein pathology in cerebellar nuclei as well as a DLB-specific atrophy in these regions^38,39^, we also included cerebellar ROIs in our analysis. Indeed, the modules in our study principally outlined the distinction between cortical and subcortical regions with the latter often clustering together with cerebellar and pontine regions. While this separation between cortical and noncortical regions into different modules was also evident in healthy controls, specifically in healthy men, men with DLB showed an even clearer separation by having one almost completely non-cortical module, except for the parahippocampal cortex. In fact, our comparisons between DLB patients and healthy controls of the same sex showed a higher local efficiency, indicating higher local integration, in men with DLB compared to healthy men. This may indicate a globally more stable grey matter network topology between healthy men and men with DLB. The main difference between healthy men and men with DLB was the emergence of a third module in DLB patients, distributing cortical regions across two modules. This may reflect a sequential neurodegeneration in cortical regions affected by the spread of the α-synuclein pathology^26^. In contrast, women groups differed in modularity, a measure that considers both local and global associations between brain regions. This suggests that the grey matter network topology in women with DLB undergo more global disorganization compared to healthy women. The remaining differences in modular organisation in women and men with DLB may diminish during the disease progress, as suggested by our previous study using univariate analyses on regional atrophy measures in the same cohort^21^.

Another module in men with DLB mainly combined frontal and posterior brain regions. The concerted decline of these brain regions is in line with functional disruptions in frontoparietal connections that were repeatedly reported in DLB patients^29^. The more disjointed combination of brain regions within and across modules, particularly the uncoupling of cerebellar regions in healthy women and the separation of pontine regions in women with DLB, aligns with the observed higher network strength in both women groups, indicating a less distinct atrophy pattern in women. Unlike in men with DLB, our modular analyses showed that the topology of atrophy we observed in women with DLB does not overlap as consistently with brain regions that have been shown to accumulate α-synuclein early in the disease process. Potentially, this less DLB-specific network disruption in women may be attributable to co-pathologies. Specifically, Alzheimer’s disease co-pathology was previously more often found in women than men with DLB^9,15^. However, comparing the positivity in both β-amyloid and tau biomarkers indicated no significant sex differences in Alzheimer’s disease co-pathology in our DLB cohort, making a sex-specific impact on grey matter networks improbable.

Some limitations should be noted. Our sample included a larger sample of DLB patients than that included in previous studies of sex differences on direct structural MRI measures in DLB^19,20^. Nonetheless, women were still underrepresented in line with the predominance of men with DLB in most European and North American DLB cohorts, from which we drew our sample^2–4^. Since the grey matter networks were based on Pearson correlation coefficients which are stabilizing with increasing sample size^40^, the smaller size of the women subsamples may have introduced some noise to their grey matter networks. However, more variance in grey matter volumes was previously reported in men compared to women^41^, which potentially balances out the effect. In addition, we equalled the number of healthy women and men to make sure our DLB findings were not influenced by having fewer women with DLB than healthy women. While the inclusion of DLB patients from different centres increases statistical power and generalizability of findings, this procedure might have introduced heterogeneity to the patient data. To counter this heterogeneity, we applied several strategies: At each centre, clinical procedures adhered to the same international guidelines and diagnostic criteria^42^. Additionally, all our statistical models accounted for centre, thus reducing the effects of inter-scanner variability. In contrast to DLB patients, data from healthy controls was derived from a single centre, potentially introducing different variability to both groups. However, using data from healthy controls from the centre providing the largest number of DLB patients in this study should have minimized this effect. Our group-based methodology did not allow us to directly assess the interaction between sex and diagnostic group in predicting network measures. While we approximated this interaction using pairwise comparisons and applying a *w*-scoring procedure, future studies might investigate such an interaction by using individual grey matter networks. With the available data for the current study, a more in-depth investigation of the relationship between grey matter network topologies and clinical features was not feasible since a further subdivision of patients with or without a specific clinical feature would have produced too small subgroups (*n* < 20) to build robust grey matter networks. Future consortia studies with larger sample sizes are necessary to investigate how the presence or absence of clinical features shapes the network topology in DLB.

Due to the cross-sectional design of our study, we cannot conclusively determine when differential sex effects first alter the grey matter networks and how changes develop over time. Longitudinal assessments of grey matter networks in patients and healthy controls are required to directly observe at which point trajectories in female and male DLB patients diverge from the trajectories of age-matched controls of the same sex.

It is worth noting that methodological choices in the construction of grey matter networks can influence outcomes^43^. The number of nodes in our grey matter networks was very similar to previous studies conducted in DLB and Alzheimer’s disease^18,44^. Given their importance in the spread of the α-synuclein pathology^26,36^, we comprehensively included non-cortical brain regions, cerebellar and pontine regions that were omitted in most previous studies^18,44^, which we consider an advantage of our study. In contrast to other studies that based their grey matter networks on covariations of regional cortical thickness and occasionally volumes of subcortical structures, we opted for a more consistent approach by exclusively considering the volumes of all included brain regions. While different morphometric parameters are associated with each other during the ageing process, their specific interactions may vary across different brain regions thus resulting in distinctive network topologies depending on which measure was employed^45^.

To conclude, our study demonstrates the dilution of sex differences in DLB, which suggests a sex-specific vulnerability of the brain network to neurodegenerative processes in DLB. For future studies, it would be of interest to determine at which point in the disease process sex-dependent trajectories start to diverge in DLB and how they progress from preclinical stages to the dementia stage. This would allow us to track the divergence of grey matter networks in women and men during healthy ageing and their convergence during the disease progression of DLB, which might be driven by an asymmetric impact of the disease on patients of one sex in particular. The dilution of sex differences in the grey matter networks of DLB patients aligns with decreasing sex differences in regional atrophy measures at older ages previously described in the same DLB cohort^21^, as well as with results from studies on functional connectivity in Parkinson’s disease^34,35^. Hence, future studies should investigate whether this feature is specific to α-synucleinopathies. Another point to be addressed in future studies will be the integration of sex-dependent trajectories in grey matter networks with sex differences observed in other biomarkers and clinical features. Our results underline the importance of considering the patient’s sex in future precision medicine approaches. While integrating sex as a factor in all clinical processes is important, it is especially crucial in diseases like DLB, in which sex differences permeate many aspects of the disease, including the vulnerability of grey matter networks, and may thus play a role in the development of treatment strategies.

## Methods

### Participants

In this cross-sectional multicentre study, we included DLB patients from three centres of the E-DLB consortium (Prague, Strasbourg, Amsterdam) as well as the Mayo Clinic DLB cohort. We previously used the same cohort to identify sex differences in regional brain volumes and cortical thickness using univariate analyses^21^. Probable DLB was diagnosed according to the 2005 International Consensus Criteria^42^. Patients were further characterized by the presence or absence of the core clinical features of DLB (parkinsonism, visual hallucinations, cognitive fluctuations, and REM sleep behaviour disorder). Additionally, performance in the Mini-Mental State Examination (MMSE) was assessed as a measure of global cognition. To assess the presence of Alzheimer’s disease (AD) co-pathology, AD biomarkers were measured in cerebrospinal fluid in E-DLB centres and with positron emission tomography at the Mayo Clinic, as described elsewhere^15^. Using centre-specific cut-points, positivity in both β-amyloid and tau biomarkers was interpreted as the presence of an AD co-pathology. Participants were excluded when they presented with any of the following: presence of acute delirium, terminal illness, previous stroke, psychotic or bipolar disorder, craniocerebral trauma, and recent diagnosis of a major somatic illness. For comparison, we included 164 sex- and age-matched, cognitively unimpaired participants from the Mayo Clinic Study of Aging (MCSA).

### Ethics declaration

The study was approved by the local ethics committee at each participating E-DLB centre and the Mayo Clinic Institutional Review Board. In compliance with the Declaration of Helsinki, all participants or appropriate surrogates provided written informed consent prior to their participation in the study.

### MRI data acquisition

A high-resolution 3D T1-weigthed magnetization-prepared rapid gradient-echo (MPRAGE) sequence was acquired in all four centres included in this study. At the Day Hospital of Geriatrics, Memory Resource and Research Centre (CMRR, Strasbourg, France), the Mayo Clinic (Rochester, US), and the VU University Medical Center (VUmc, Amsterdam, the Netherlands), images were acquired at a magnetic field strength of 3 T, whereas the Motol University Hospital (Prague, Czech Republic) used a 1.5 T scanner. Additional details on scanning parameters in each centre are provided in the supplement.

### MRI preprocessing

All MRI data were processed at the Mayo Clinic, following previously detailed procedures^46^. Briefly, using Advanced Normalization Tools (ANTs), the Mayo Clinic Adult Lifespan Template (MCALT; https://www.nitrc.org/projects/mcalt/) atlas was registered to individuals’ native MPRAGE space. T1-MPRAGE images were then tissue-class segmented using the unified segmentation algorithm in SPM12 (Wellcome Trust Center for Neuroimaging, London, UK) run in Matlab (Mathworks, Natick, MA), with priors and settings from the MCALT. Following MCALT parcellation, we obtained the volumes of 58 grey matter regions-of-interest (ROIs), consisting of 41 cortical, 6 subcortical, 9 cerebellar (as the sum of both hemispheres), and 2 brainstem ROIs, for each participant. Additionally, the total intracranial volume (TIV) was calculated as the sum of tissue probabilities of grey matter, white matter, and cerebrospinal fluid segmentations.

### Network construction and analysis

Grey matter networks were constructed from the volumetric data of the 58 ROIs. For aim 1, ROI data was adjusted for TIV^47^ and centre (which also removes the variability associated to differences in MRI field strength), using multiple linear regression prior to network construction. For aim 2, which addressed DLB-specific sex differences in grey matter networks independent of sex differences in healthy ageing, we regressed out the expected effect of TIV, age, and sex found in the matched healthy controls by calculating *w*-scores prior to network construction. For each ROI, the following formula (1) was applied:

1 $$\begin{array}{l}{w{{\mbox{-}}}{score}}_{{DLB\; patient}}\\=\frac{{{raw\; value}}_{{DLB\; patient}}-{{expected\; value}}_{{healthy\; control}}\,\left({for\; patien}{t}^{{\prime} }{s\; TIV},{age},{sex}\right)}{{{SD\; of\; residuals}}_{{healthy\; controls}}}\end{array}$$

*W*-scored DLB data were additionally corrected for centre, using linear regression. For aim 3, ROI data was adjusted for TIV and centre.

We then used the outcome from these adjustments to construct separate grey matter networks for each sex for DLB patients, healthy controls, and *w*-scored DLB data. Therein, nodes correspond to the residual volumes of the 58 ROIs and edges represent the Pearson correlation coefficients between each node pair. Self-connections along the diagonal were removed from the correlation matrices. While compensatory functional processes have been reported in DLB^48^, we did not expect compensatory structural processes and thus also excluded negative correlations from the grey matter networks.

We calculated several measures to define the centrality, integration, and segregation of the grey matter networks, both on the global (averaging across all nodes) and nodal (concerning single nodes) level^49^. Nodal strength (measure of centrality, sum of all connections of a node) was calculated on the weighted correlation matrices. All other network measures were calculated on unweighted binarized networks, which were constructed by thresholding networks at densities between 23% to 64% in steps of 1%. After removal of the 4 least connected nodes (caudate, pallidum, putamen, and MCALT atlas region 10 of cerebellum) in grey matter networks of DLB patients and healthy controls, this procedure ensured that the networks were connected, with each node being connected to at least one other node (network density >23%), and exhibited a non-random network topology, with a small-world index >1.2 in relation to a random symmetrical network without self-connections (network density <64%). Sex differences in grey matter network parameters were assessed across this range of network densities. We calculated the following global network measures on the grey matter networks: global efficiency (measure of integration, reciprocal of the node’s shortest path lengths to every other node), local efficiency (measure of segregation, reciprocal of a node’s shortest path length in the subgraph of the node’s neighbours), modularity (measure of integration and segregation, extent to which a network can be divided into distinct modules), transitivity (measure of segregation, fraction of a node’s neighbours that are neighbours of each other), and betweenness centrality (measure of centrality, number of shortest paths in the network that traverse a given node)^49^. To pinpoint specific nodes whose status in the network differed between sexes, we additionally computed the following nodal network measures: nodal global efficiency, nodal local efficiency, and nodal betweenness centrality. While multiple network measures are available^49^, we based our choice on the measures that have been reported to be more stable^50^.

While the modularity measure allows the quantification of the degree to which distinct nodes aggregate into more densely connected modules, it does not allow any insights on the topology of those modules. Therefore, to describe modules in each of the groups qualitatively, we conducted modular analyses on the weighted correlation matrices using the Newman algorithm^51^.

All network analyses were conducted in Matlab R2019b using customized scripts based on the Brain Connectivity Toolbox version 2019-03-03^49^.

### Statistical analysis

Group differences in demographic and clinical variables were checked with *t*-tests, one-way ANOVAs, and Fisher’s exact tests (all two-tailed) for between-group comparisons of continuous and categorical variables, respectively. An α-level of *p* < 0.05 (two-tailed) denoted statistical significance. Whenever an ANOVA showed a significant group effect, we conducted post-hoc *t*-tests between all four groups, applying the Bonferroni correction. Between-group comparisons of network measures were conducted through 10,000 nonparametric permutations at a range of network densities (23–64%, in steps of 1%). Again, the significance threshold was set to *p* < 0.05 (two-tailed) for global network measures. Global measures with significant differences in ≥5 network densities were considered significant. For nodal measures, an additional false discovery rate (FDR) adjustment for multiple comparisons was applied at *p* < 0.05 (two-tailed) at all network densities. Nodal measures surviving FDR correction for ≥5 network densities were considered significant. All 58 nodes were included in the main network analyses. To test the robustness of these results, we repeated the analyses of global network measures after removing the 4 least connected nodes (caudate, pallidum, putamen, and MCALT atlas region 10 of cerebellum).

### Reporting summary

Further information on research design is available in the Nature Research Reporting Summary linked to this article.

### Supplementary information

Supplementary Material

Reporting Summary

## Data Availability

The data that support the findings of this study are available through the E-DLB consortium (https://www.e-dlb.com) and the Mayo Clinic (https://www.mayo.edu/research/labs/aging-dementia-imaging/overview) for qualified researchers upon request.

## Code availability

The underlying code for this study is not publicly available but may be made available to qualified researchers upon request to the corresponding author.
