# Supplementary material for: Grey matter networks in women and men with dementia with Lewy bodies
Source: NPJ Parkinsons Dis. 2024 Apr 13;10:84. doi: 10.1038/s41531-024-00702-5 (PMC11016082; doi:10.1038/s41531-024-00702-5)
Supplement: Supplementary file 1 — Supplementary Material [file 41531_2024_702_MOESM1_ESM.pdf]

Supplementary material

	MODULE AFFILIATION					
	HEALTHY CONTROLS		DLB PATIENTS		DLB PATIENTS (*w*-scores)	
**BRAIN REGIONS**	**Women**	**Men**	**Women**	**Men**	**Women**	**Men**
**Amygdala**	3	2	3	1	3	3
**Angular**	1	2	2	1	2	3
**Calcarine**	1	1	1	1	1	3
**Caudate**	3	2	2	2	2	2
**Cingulum Ant**	1	1	1	1	3	3
**Cingulum Mid**	1	1	1	1	3	3
**Cingulum Post**	1	2	1	1	3	3
**Cuneus**	1	1	1	1	1	3
**Entorhinal Cortex**	3	2	3	3	3	1
**Frontal Inf Oper**	3	2	1	3	3	1
**Frontal Inf Orb**	3	1	1	3	3	1
**Frontal Inf Tri**	3	1	1	1	3	3
**Frontal Med Orb**	1	1	3	3	3	1
**Frontal Mid Orb**	2	1	3	3	3	1
**Frontal Mid**	1	2	1	1	3	3
**Frontal Sup Medial**	1	1	3	1	3	3
**Frontal Sup Orb**	1	1	3	3	3	1
**Frontal Sup**	3	1	3	1	3	3
**Fusiform**	1	2	1	3	3	1
**Heschl**	1	1	1	1	1	3
**Hippocampus**	3	2	3	2	3	2
**Insula**	1	1	1	3	3	1
**Lingual**	1	1	1	1	1	3
**Occipital Inf**	1	1	1	1	1	3
**Occipital Mid**	1	2	1	1	1	3
**Occipital Sup**	1	2	1	1	1	3
**Olfactory**	3	1	1	3	3	3
**Pallidum**	3	2	2	2	2	2
**ParaHippocampal**	1	2	1	2	1	2
**Paracentral Lobule**	3	1	1	3	1	1
**Parietal Inf**	1	2	1	1	3	3
**Parietal Sup**	1	2	1	1	3	3
**Postcentral**	1	1	1	3	1	1
**Precentral**	1	1	1	3	1	1
**Precuneus**	1	1	1	1	3	3
**Putamen**	3	2	2	2	2	2
**Rectus**	1	1	3	3	3	1
**Retrosplenial Cortex**	1	2	1	3	1	1
**Rolandic Oper**	1	1	1	3	1	1
**Supp Motor Area**	3	1	3	3	1	1
**SupraMarginal**	1	1	1	3	3	1
**Temporal Inf**	3	1	1	3	3	1
**Temporal Mid**	1	1	1	3	1	1
**Temporal Pole Mid**	3	1	3	3	3	1
**Temporal Pole Sup**	3	1	3	3	3	1
**Temporal Sup**	1	1	1	3	3	1
**Thalamus**	2	2	1	2	1	2

**Cerebellum 10**	2	2	2	2	2	2
**Cerebellum 3**	2	2	2	2	2	2
**Cerebellum 4 5**	2	2	2	2	2	2
**Cerebellum 6**	2	2	2	2	2	2
**Cerebellum 7b**	3	2	2	2	2	2
**Cerebellum 8**	3	2	2	2	2	2
**Cerebellum 9**	2	2	2	2	2	2
**Cerebellum Crus1**	2	2	2	2	2	2
**Cerebellum Crus2**	3	2	2	2	2	2
**Pons**	2	2	2	2	2	2
**Dorsal Mesopontine**	2	2	1	2	2	2

Supplementary table 1: Module affiliation for each brain region for women and men healthy controls, DLB patients, and *w*-scores. Numbers in the table refer to modules 1, 2, and 3 in figure 3 of the main manuscript, respectively.

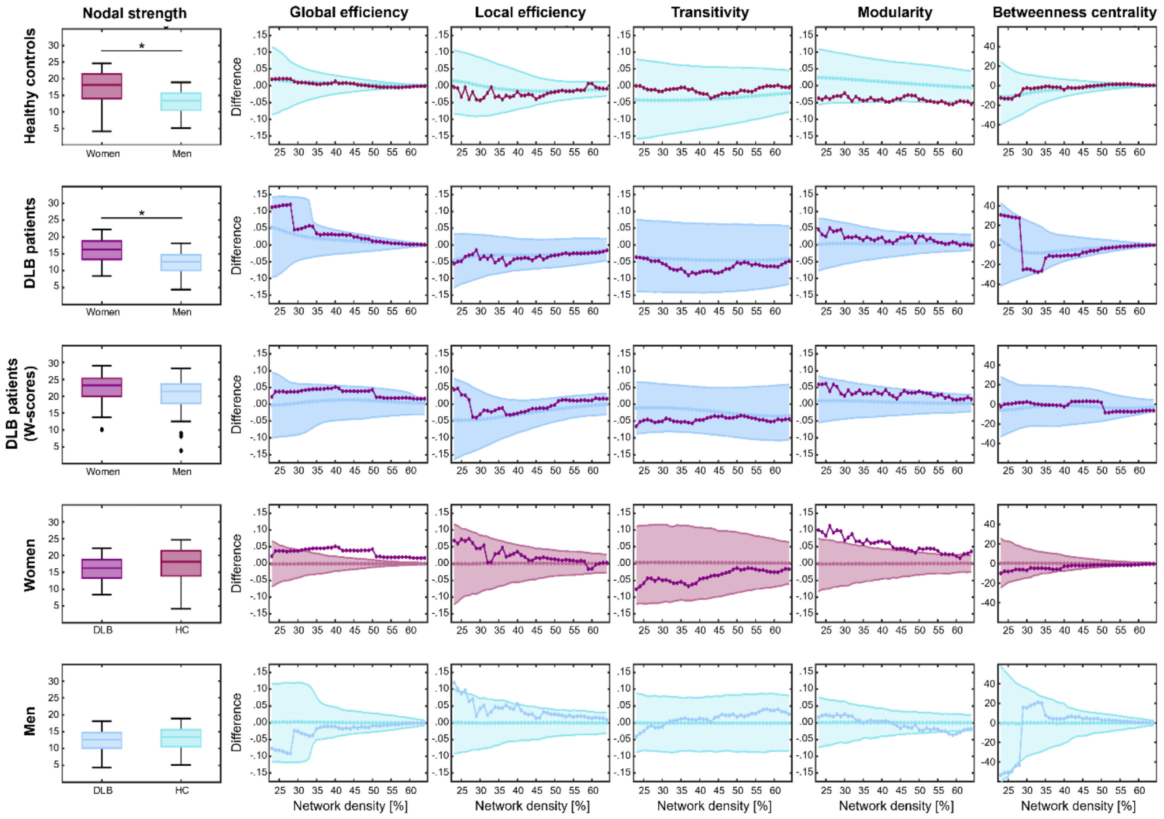

Supplementary figure 1: Sensitivity analyses. Comparison of global network measures after removal of the 4 least connected nodes (caudate, pallidum, putamen, and MCALT atlas region 10 of cerebellum). For nodal strength, box limits denote 25th and 75th percentiles, while whiskers indicate extreme data points without outliers. The central line denotes the median. For the remaining network measures, grey matter network densities are displayed on the x-axis from min=23% to max=64%, in steps of 1%. Group differences are displayed on the y-axis with 95% confidence intervals of 10’000 permutations. Negative differences indicate lower value in women or DLB patients compared to men or healthy controls, respectively. Positive differences indicate higher values in women or DLB patients compared to men or healthy controls, respectively. DLB = dementia with Lewy bodies. HC = healthy controls.
