# Supplementary material for: Grey matter networks in women and men with dementia with Lewy bodies
Source: NPJ Parkinsons Dis. 2024 Apr 13;10:84. doi: 10.1038/s41531-024-00702-5 (PMC11016082; doi:10.1038/s41531-024-00702-5)
Supplement: Supplementary file 2 — Reporting Summary [file 41531_2024_702_MOESM2_ESM.pdf]

nature portfolio

Corresponding author(s): Daniel Ferreira

Last updated by author(s): Mar 26, 2024

Reporting Summary

Nature Portfolio wishes to improve the reproducibility of the work that we publish. This form provides structure for consistency and transparency in reporting. For further information on Nature Portfolio policies, see our Editorial Policies and the Editorial Policy Checklist.

Statistics

For all statistical analyses, confirm that the following items are present in the figure legend, table legend, main text, or Methods section.

n/a	Confirmed	
☐	☒	The exact sample size (*n*) for each experimental group/condition, given as a discrete number and unit of measurement
☐	☒	A statement on whether measurements were taken from distinct samples or whether the same sample was measured repeatedly
☐	☒	The statistical test(s) used AND whether they are one- or two-sided<br>*Only common tests should be described solely by name; describe more complex techniques in the Methods section.*
☐	☒	A description of all covariates tested
☐	☒	A description of any assumptions or corrections, such as tests of normality and adjustment for multiple comparisons
☐	☒	A full description of the statistical parameters including central tendency (e.g. means) or other basic estimates (e.g. regression coefficient) AND variation (e.g. standard deviation) or associated estimates of uncertainty (e.g. confidence intervals)
☐	☒	For null hypothesis testing, the test statistic (e.g. *F*, *t*, *r*) with confidence intervals, effect sizes, degrees of freedom and *P* value noted<br>*Give P values as exact values whenever suitable.*
☒	☐	For Bayesian analysis, information on the choice of priors and Markov chain Monte Carlo settings
☒	☐	For hierarchical and complex designs, identification of the appropriate level for tests and full reporting of outcomes
☒	☐	Estimates of effect sizes (e.g. Cohen's *d*, Pearson's *r*), indicating how they were calculated

Our web collection on statistics for biologists contains articles on many of the points above.

Software and code

Policy information about availability of computer code

Data collection	N/A
Data analysis	Matlab R2019b (Mathworks, Natick, MA); Brain Connectivity Toolbox version 2019-03-03

For manuscripts utilizing custom algorithms or software that are central to the research but not yet described in published literature, software must be made available to editors and reviewers. We strongly encourage code deposition in a community repository (e.g. GitHub). See the Nature Portfolio guidelines for submitting code & software for further information.

Data

Policy information about availability of data

All manuscripts must include a data availability statement. This statement should provide the following information, where applicable:

- Accession codes, unique identifiers, or web links for publicly available datasets
- A description of any restrictions on data availability
- For clinical datasets or third party data, please ensure that the statement adheres to our policy

Data from the E-DLB consortium ((https://www.edlb.com) and the Mayo Clinic (https://www.mayo.edu/research/labs/aging-dementia-imaging/overview) will be made available to qualified researchers upon request to the corresponding author.

Research involving human participants, their data, or biological material

Policy information about studies with human participants or human data. See also policy information about sex, gender (identity/presentation), and sexual orientation and race, ethnicity and racism.

Reporting on sex and gender	Analyses of sex differences are the principal objective of this study and thus an inherent part of the study design. All sex-related analyses are reported in the manuscript.<br>A total of 45 women and 119 men with DLB, as well as 45 healthy women and 119 healthy men (sex- and age-matched) were included in the study.
Reporting on race, ethnicity, or other socially relevant groupings	No data on race or ethnicity was available for this study.
Population characteristics	164 DLB patients from three centres of the E-DLB consortium (Prague, Strasbourg, Amsterdam) as well as the Mayo Clinic DLB cohort (Rochester, USA) along with 164 healthy age- and sex-matched controls from the Mayo Clinic Study of Aging (MCSA) were included in this study. Probable DLB was diagnosed according to the 2005 International Consensus Criteria.22 Patients were further characterized by the presence or absence of the core clinical features of DLB (parkinsonism, visual hallucinations, cognitive fluctuations, and REM sleep behaviour disorder). Additionally, performance in the Mini-Mental State Examination (MMSE) was assessed as a measure of global cognition. To assess the presence of Alzheimer's disease (AD) co-pathology, AD biomarkers were measured in cerebrospinal fluid in E-DLB centres and with positron emission tomography at the Mayo Clinic, as described elsewhere.15 Using centre-specific cut-points, positivity in both β-amyloid and tau biomarkers was interpreted as the presence of an AD co-pathology.
Recruitment	Please see above.
Ethics oversight	The study was approved by the local ethics committee at each participating E-DLB centre and the Mayo Clinic Institutional Review Board. In compliance with the Declaration of Helsinki, all participants or appropriate surrogates provided written informed consent prior to their participation in the study.

Note that full information on the approval of the study protocol must also be provided in the manuscript.

Field-specific reporting

Please select the one below that is the best fit for your research. If you are not sure, read the appropriate sections before making your selection.

☒ Life sciences ☐ Behavioural & social sciences ☐ Ecological, evolutionary & environmental sciences

For a reference copy of the document with all sections, see nature.com/documents/nr-reporting-summary-flat.pdf

Life sciences study design

All studies must disclose on these points even when the disclosure is negative.

Sample size	164 DLB patients from three centres of the E-DLB consortium (Prague, Strasbourg, Amsterdam) as well as the Mayo Clinic DLB cohort (Rochester, USA) along with 164 healthy age- and sex-matched controls from the Mayo Clinic Study of Aging (MCSA) were included in this study. All DLB patients with available structural MRI data and baseline MMSE were included in this study. Healthy control participants were matched by age and sex to the DLB patients.
Data exclusions	Participants without structural MRI scans or MMSE to assess global cognition were excluded from the study. Participants were excluded when they presented with any of the following: presence of acute delirium, terminal illness, previous stroke, psychotic or bipolar disorder, craniocerebral trauma, and recent diagnosis of a major somatic illness.
Replication	Participants without structural MRI scans or MMSE to assess global cognition were excluded from the study. Participants were excluded when they presented with any of the following: presence of acute delirium, terminal illness, previous stroke, psychotic or bipolar disorder, craniocerebral trauma, and recent diagnosis of a major somatic illness.
Randomization	Randomization was not relevant for the study since the study had a retrospective cross-sectional design.
Blinding	Blinding was not relevant for the study since no intervention was carried out.

Reporting for specific materials, systems and methods

We require information from authors about some types of materials, experimental systems and methods used in many studies. Here, indicate whether each material, system or method listed is relevant to your study. If you are not sure if a list item applies to your research, read the appropriate section before selecting a response.

Materials & experimental systems

n/a	Involved in the study
☒	☐ Antibodies
☒	☐ Eukaryotic cell lines
☒	☐ Palaeontology and archaeology
☒	☐ Animals and other organisms
☒	☐ Clinical data
☒	☐ Dual use research of concern
☒	☐ Plants

Methods

n/a	Involved in the study
☒	☐ ChIP-seq
☒	☐ Flow cytometry
☐	☒ MRI-based neuroimaging

Plants

Seed stocks

Report on the source of all seed stocks or other plant material used. If applicable, state the seed stock centre and catalogue number. If plant specimens were collected from the field, describe the collection location, date and sampling procedures.

Novel plant genotypes

Describe the methods by which all novel plant genotypes were produced. This includes those generated by transgenic approaches, gene editing, chemical/radiation-based mutagenesis and hybridization. For transgenic lines, describe the transformation method, the number of independent lines analyzed and the generation upon which experiments were performed. For gene-edited lines, describe the editor used, the endogenous sequence targeted for editing, the targeting guide RNA sequence (if applicable) and how the editor was applied.

Authentication

Describe any authentication procedures for each seed stock used or novel genotype generated. Describe any experiments used to assess the effect of a mutation and, where applicable, how potential secondary effects (e.g. second site T-DNA insertions, mosiacism, off-target gene editing) were examined.

Magnetic resonance imaging

Experimental design

Design type

Sructural MRI T1-based data.

Design specifications

N/A

Behavioral performance measures

N/A

Acquisition

Imaging type(s)

Structural T1-MRI

Field strength

1.5T (Prague); 3T(Amsterdam, Strasbourg, Mayo Clinic)

Sequence & imaging parameters

Motol University Hospital (Prague): Siemens Avanto, T1-weighted, TR=2000, TE3.08
VU University Medical Center (VUmc, Amsterdam): Discovery 750MR, T1-weighted, TR=8, TR=3
Day Hospital of Geriatrics, Memory Resource and Research Centre (CMRR, Strasbourg): Siemens Verio, T1-weighted, TR=1900, TE=2.53
Mayo Clinic (Rochester, US): Discovery 750 MR, T1-weighted, TR=2300, TE=3

Area of acquisition

Whole brain

Diffusion MRI ☐ Used ☒ Not used

Preprocessing

Preprocessing software

All MRI data were processed at the Mayo Clinic, following previously detailed procedures.(1) Briefly, using Advanced Normalization Tools (ANTs), the Mayo Clinic Adult Lifespan Template (MCALT; https://www.nitrc.org/projects/mcalt/) atlas was registered to individuals' native MPRAGE space. T1-MPRAGE images were then tissue-class segmented using the unified segmentation algorithm in SPM12 (Wellcome Trust Center for Neuroimaging, London, UK) run in Matlab (Mathworks, Natick, MA), with priors and settings from the MCALT. Following MCALT parcellation, we obtained the volumes of 58 grey matter regions-of-interest (ROIs), consisting of 41 cortical, 6 subcortical, 9 cerebellar (as the sum of both hemispheres), and 2 brainstem ROIs, for each participant. Additionally, the total intracranial volume (TIV) was calculated as the sum of tissue probabilities of grey matter, white matter, and cerebrospinal fluid segmentations.

References:
(1) Schwarz CG, Gunter JL, Wiste HJ, et al. A large-scale comparison of cortical thickness and volume methods for measuring Alzheimer's disease severity. NeuroImage: Clinical. 2016;11:802–812.

Normalization | Specifications above (Section: Preprocessing software)
Normalization template | Specifications above (Section: Preprocessing software)
Noise and artifact removal | Specifications above (Section: Preprocessing software)
Volume censoring | Specifications above (Section: Preprocessing software)

Statistical modeling & inference

Model type and settings

Group differences in demographic and clinical variables were checked with t-tests, one-way ANOVAs, and Fisher's exact tests for between-group comparisons of continuous and categorical variables, respectively. An α-level of $p< 0.05$ (two-tailed) denoted statistical significance. Whenever an ANOVA showed a significant group effect, we conducted post-hoc t-tests between all four groups, applying the Bonferroni correction. Between-group comparisons of network measures were conducted through 10000 nonparametric permutations at a range of network densities (23% - 64%, in steps of 1%). Again, the significance threshold was set to $p<0.05$ (2-tailed) for global network measures. Global measures with significant differences in ≥5 network densities were considered significant. For nodal measures, an additional false discovery rate (FDR) adjustment for multiple comparisons was applied at $p < 0.05$ (two-tailed) at all network densities. Nodal measures surviving FDR correction for ≥ 5 network densities were considered significant. All 58 nodes were included in the main network analyses. To test the robustness of these results, we repeated the analyses of global network measures after removing the 4 least connected nodes (caudate, pallidum, putamen, and MCALT atlas region 10 of cerebellum).

Effect(s) tested

Sex differences were assessed comparing healthy women and healthy men, women with DLB and men with DLB (with additional analyses based on w-scored networks of DLB patients to remove sex differences associated with healthy ageing). Aditionally, we assessed differences between women with DLB and healthy women as well as between men with DLB and healthy men

Specify type of analysis: ☐ Whole brain ☒ ROI-based ☐ Both

Anatomical location(s)

Brain regions defined in the Mayo Clinic Adult Lifespan Template (MCALT) were used to construct the grey matter networks. We included volumes of 58 grey matter regions-of-interest (ROIs), consisting of 41 cortical, 6 subcortical, 9 cerebellar (as the sum of both hemispheres), and 2 brainstem ROI.

Statistic type for inference

(See Eklund et al. 2016)

N/A

Correction

For nodal measures, an additional false discovery rate (FDR) adjustment for multiple comparisons was applied at $p < 0.05$ (two-tailed) at all network densities.
For nodal measures, an additional false discovery rate (FDR) adjustment for multiple comparisons was applied at $p < 0.05$ (two-tailed) at all network densities

Models & analysis

n/a	Involved in the study
☒	☐ Functional and/or effective connectivity
☐	☒ Graph analysis
☒	☐ Multivariate modeling or predictive analysis

Graph analysis

Grey matter networks were constructed based on group-level pair-wise correlations between the volumes of the 58 ROIs. Nodal strength was calculated on the weighted matrices. All other network measures were calculated on binarized matrices, thresholded at a range of network densities (23% - 64%, in steps of 1%).
The following global network measures were calculated on the grey matter networks: global efficiency (measure of integration, reciprocal of the node's shortest path lengths to every other node), local efficiency (measure of segregation, reciprocal of a node's shortest path length in the subgraph of the node's neighbours), modularity (measure of integration and segregation, extent to which a network can be divided into distinct modules), transitivity (measure of segregation, fraction of a node's neighbours that are neighbours of each other), and betweenness centrality (measure of centrality, number of shortest paths in the network that traverse a given node).
Additionally, we calculated the following nodal network measures: nodal global efficiency, nodal local efficiency, and nodal betweenness centrality.
